# Proteus Syndrome, Anticipated Difficult Airway, and 2 Trusted Lieutenants!

**DOI:** 10.5152/TJAR.2021.21149

**Published:** 2022-12-01

**Authors:** Vansh Priya, Rafat Shamim, Ganpat Prasad

**Affiliations:** Department of Anaesthesia, Sanjay Gandhi Postgraduate Institute of Medical Sciences, Lucknow, India

Proteus syndrome caused by mosaic variant in a gene called AKT1 results in asymmetric overgrowth of various tissues of the body. It may affect bone and connective tissue, fatty tissues, skin, central nervous system, and internal organs. A 2-year-old male child presented with gross swelling of left face and was listed for debulking surgery (Figure 1A). Magnetic resonance imaging revealed thickening of left hemifacial bones, marked lipomatous hypertrophy involving subcutaneous tissue, temporal, infratemporal, parapharyngeal space, floor of mouth, and base of neck (Figure 2). Airway evaluation showed restricted and deviated mouth opening with disorganised dentition of left side and Mallampati grade IV. Child was premedicated with intranasal dexmedetomidine and shifted to operating room. For optimum bag and mask ventilation and laryngoscopy, child was placed in back up and head elevated position (Figure 1B). For preserving spontaneous ventilation and achieving adequate depth of anaesthesia using inhalation induction with sevoflurane, intravenous fentanyl (1 µg kg^−1^) was administered. Check video laryngoscopy using Glidescope for molar approach to intubation was performed and Cormack Lehane grade II was observed. Inj Rocuronium (1 mg kg^−1^) was administered, and the child was intubated using uncuffed tube of size 4.5 (Figure 3). Back up head elevated position is known to improve laryngoscopy, prolong safe apnoea time, and reduce complications in anticipated difficult airway.^1^ Right molar approach to intubation facilitates intubation particularly in intraoral mass, by reducing distance from the patient’s teeth to the larynx and thus preventing intrusion of intraoral, maxillary structures into the line of vision thereby obviating the need for alignment of oropharyngeal and laryngeal axis.^2^ There is still lack of guidelines for management of anticipated paediatric difficult airway. Lack of co-operation in this age group rules out awake fibreoptic bronchoscopy-guided intubation and awake tracheal intubation. Further in this case, distorted upper airway anatomy ruled out the use of supraglottic airway.

## Figures and Tables

**Figure 1.**
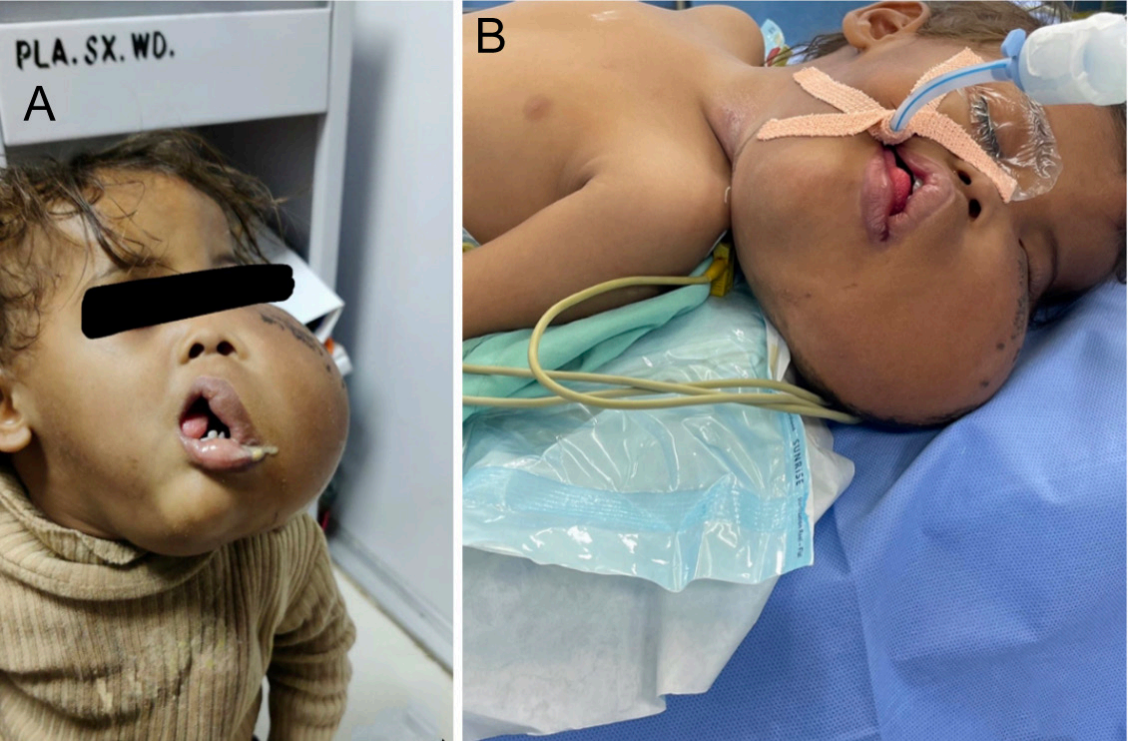
(A) A 2-year-old male child with Proteus syndrome listed for debulking surgery. (B) Back up head elevated position of child for laryngoscopy and intubation.

**Figure 2.**
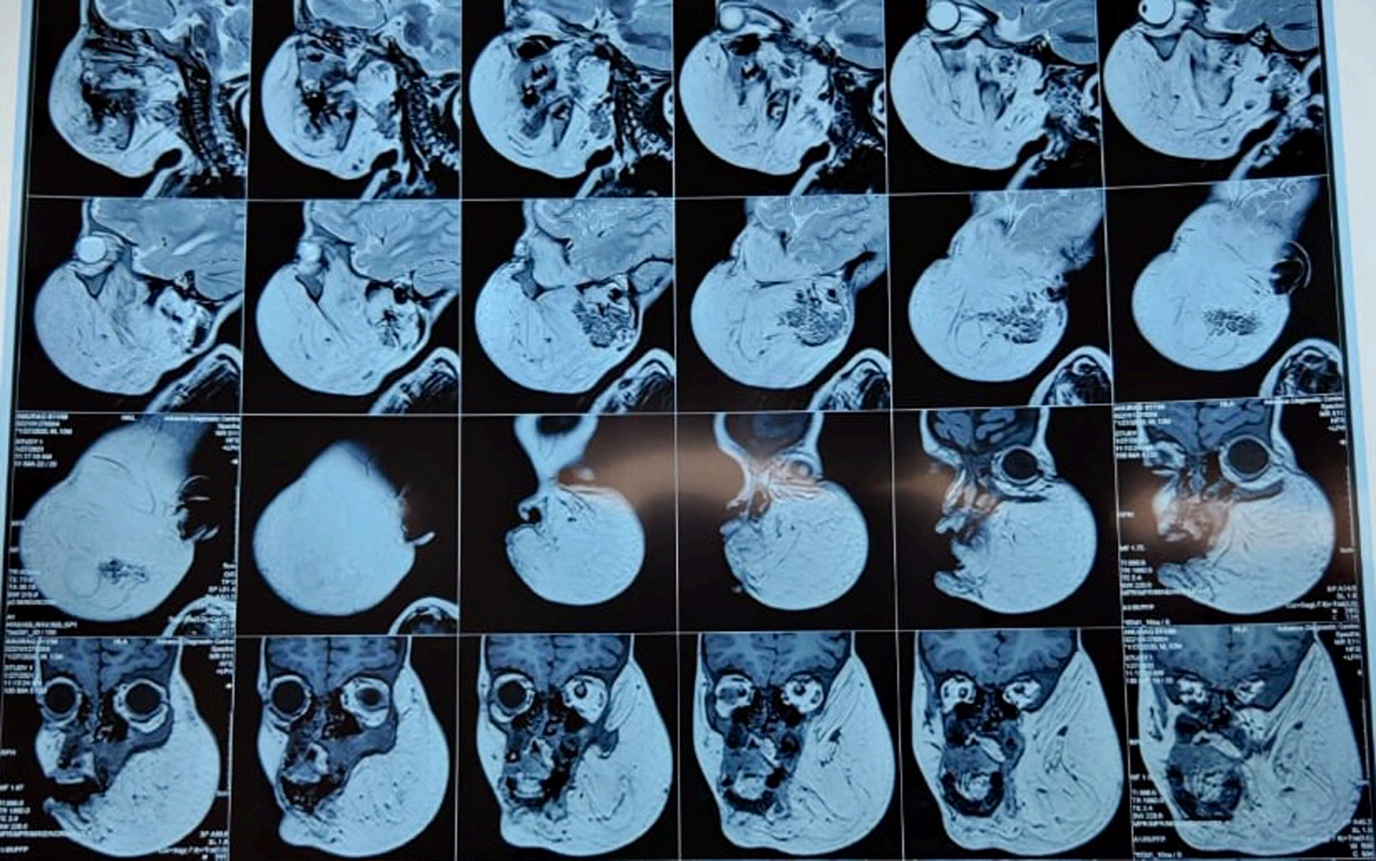
Magnetic resonance imaging scan.

**Figure 3.**
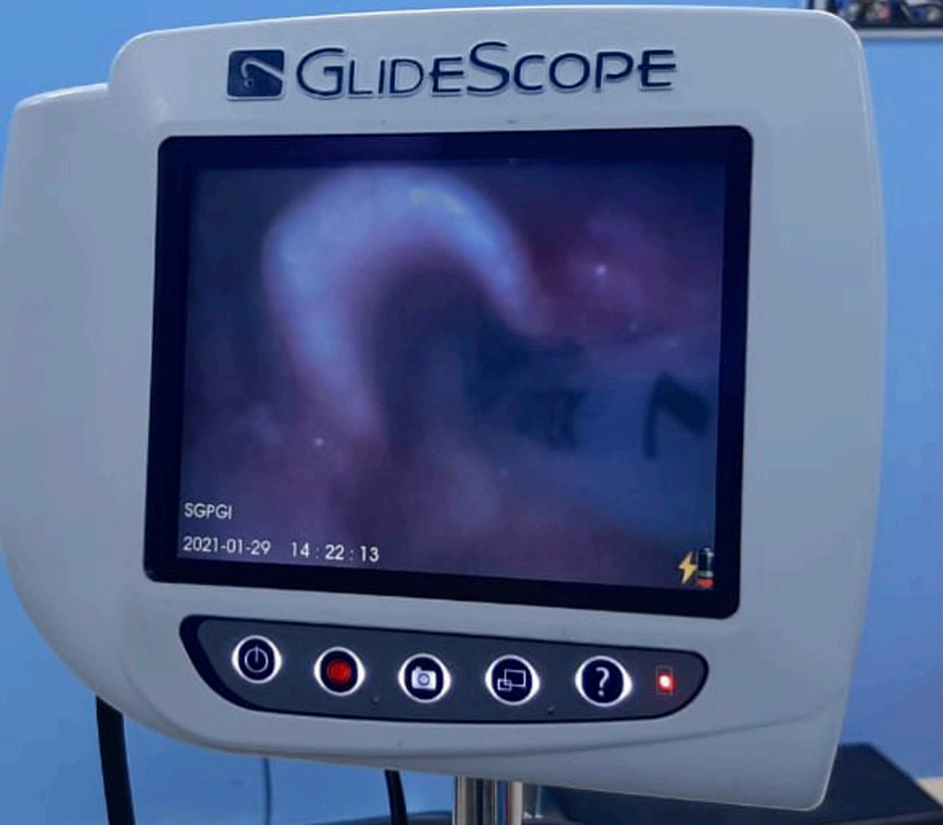
Use of Glidescope^®^ video laryngoscope to intubate.
